# Heading towards an unsustainable world: some of the implications of not achieving the SDGs

**DOI:** 10.1007/s43621-020-00002-x

**Published:** 2020-09-04

**Authors:** Walter Leal Filho, Franziska Wolf, Amanda Lange Salvia, Ali Beynaghi, Kalterina Shulla, Marina Kovaleva, Claudio R. P. Vasconcelos

**Affiliations:** 1 European School of Sustainability Science and Research, Hamburg University of Applied Sciences, Ulmenliet 20, 21033 Hamburg, Germany; 2 Office of Sustainability, Amirkabir University of Technology (Tehran Polytechnic), Tehran, Iran; 3 ZEF, Center for Development Research, University of Bonn, Germany, Genscherallee 3, 53113 Bonn, Germany; 4 Laboratory of Sustainability Engineering and Consumption, Federal University of Paraíba, João Pessoa, PB Brazil; 5 Algoritmi Research Centre, School of Engineering, University of Minho, 4800-058 Guimarães, Portugal

## Abstract

The Sustainable Development Goals (SDGs) were conceived at the United Nations Conference on Sustainable Development, held in Rio de Janeiro in 2012 (Rio + 20), and adopted by the United Nations General Assembly in September 2015. They are part of a larger framework, namely the UN 2030 Agenda for Sustainable Development. Since then, many countries round the world have been engaging in respect of their implementation. The slow progress seen in the implementation of the SDGs, is in contrast with the many negative implications of not implementing them. This paper outlines the relevance of the SDGs, the barriers currently seen in respect of their implementation and outlines what is at stake, if they are not duly implemented. To accomplish this, a thorough literature review of contributions published in the field of SDGs in English between the years 2012–2020 was performed.

## Introduction: the importance of the Sustainable Development Goals

During the past centuries, almost all the systems have been developed through natural processes. However, with the technological advancements today, development is moving faster than our expectations. Therefore, having no plan for well-being and environment can cause serious problems to the future societies. As a result of the fast-moving unbalanced economic growth, the world’s systems may lose their ability to adjust with the people’s well-being which also significantly affect the environment. In this era, having a systematic action plan can definitely help to focus on a common perspective.

To this end, on 25 September 2015, countries in UN adopted a set of 17 Sustainable Development Goals (SDGs), representing a new coherent way of thinking about ecological, social, and economic issues that are inter-related [1]. Considering as the “transformative agenda” [2], the SDGs address all the critical and major global challenges that threat the future, including those related to poverty, inequality, climate change, environmental degradation, peace and justice, necessarily entails an integrated approach.

Different studies have analyzed challenges and opportunities of the SDGs [3, 4]. Some scholars and practitioners have referred to the fact that these goals are not easy to achieve due to the diversity of the elements involved [5]. Moreover, the importance of including interdependencies between the goals [6, 7], taking a nexus approach [8–10], and strengthening governance and institutions [11] have been highlighted in some studies in order to achieve these goals. Nevertheless, the significance of the SDGs can be viewed from different perspectives. For instance:

- *Addressing essential human needs.* As the world population grows fast, there is an essential need for the basics of life such as drinking water, food, and shelter. To achieve this, appropriate establishment of plans as well as creating infrastructures are able to guarantee the sustainability of these basic needs for long term periods [10].
- *Managing climate change.* Strategies for sustainable development and climate change have shown many common fundamentals, suggesting that sustainable development is a key to capacities for mitigation and adaptation [12]. It is believed that addressing them jointly can synergistically help the future generations. This is one of the main purposes of the SDGs to reduce the excessive use of harmful fossil-based sources. To take the example of China, air pollution from over-reliance on coal-based electricity generation and other fossil fuels contributes to the deaths of more than 1.2 million per year [13]. Therefore, such a path is not merely unsustainable; it is a prescription for disaster. Hence, the 2030 Agenda and its 17 SDGs highlight that a sustainable transformation to renewable energies is sorely needed [14, 15].
- *Financial stability.* Financing has always played a key role in the realization of other targets and goals. Sustainable development practices can potentially have a decisive influence on the stability of the financial system to create more financially sustainable economies across the globe. The critical role of energy has been highlighted in this road by the former United Nations Secretary-General Ban Ki-moon: “energy is the golden thread that connects economic growth, social equity, and environmental sustainability” [16]. As a result of the low carbon policy, developing countries can leverage renewable forms of energy to compete with the developed countries and to power up their economies. It is believed that by achieving the SDGs in this sector, many sustainable jobs will be created, affecting the stability of the economy [17].
- *Sustaining biodiversity.* The current state of overconsumption practices all around the world has negatively affected biodiversity. The natural ecosystem is designed in a subsequent manner that species depend on one another in a circular way. The SDGs and affords to achieving them encourage us to back to the nature. This will result a balance in the biodiversity of different species [18–20].

Finally, it should be noted that to achieve the SDGs, based on the multidisciplinary nature of sustainability: (1) Innovative transition pathways are required to allow countries to move forward, and (2) a collaborative work should be done in a global scale. Focusing on universality of the 2030 agenda, although countries develop their own pathways to national targets, it is not limited to the borders and cultures. The ultimate goal of all these affords is saving the whole planet for the future generations. Hereafter, the paper discusses barriers to the implementation of the SDGs, followed by the used methodology, and results and discussion, presenting some of the consequences of not reaching the SDG with an emphasis on a worst-case scenario.

## Barriers to the implementation of the UN Sustainable Development Goals

Although the SDGs, also known as “global goals” are supported by several countries, many challenges current hinder their implementation. Some of them are as follows:

1. Vague goals: Several of the goals are not well explained allowing for own interpretation and thus weak implementation. Therefore, it is imperative that the SDGs have indicators and benchmarks as well as formal agreements with governments for implementation [21].
2. Collective action: It is difficult to ensure that several parties work together to achieve the set goals. Several coordination issues exist preventing the efficient implementation of goals [22].
3. Trade-offs: The 17 SDGs were developed to ensure economic and environmental sustainability however, conflicts between each goal may exist. This causes the need for trade-offs to be identified i.e. sacrificing parts of one goal to benefit another goal when it is impossible to achieve both. Until all trade-offs and conflicts are identified it will be difficult to implement goals [23].
4. Accountability: A major challenge in application of goals is ensuring accountability for commitments made to the SDGs. This will ensure that targets and goals will be met within the prescribed period of time by the committed parties. A lack of accountability will reduce the chances of proper implementation [24, 25].
5. Financial constraints: In order to achieve goals set, a large amount of financial investment is required. Several countries lack the capital needed to fund the programmes involved in reaching the SDGs. This is more evident in third world countries as opposed to developed countries [26].
6. Capacity building: This requires parties involved in the development programmes to acquire all the skills, tools and education needed to carry out tasks to achieve goals. This is often not possible due to various reasons including location, finance, and trained personnel [26].
7. Technology and data: The success of any project requires the large-scale collection of data and monitoring of trends. This process is dependent on having the necessary updated technology which is not always available. This is more so apparent in developing countries making it more difficult to implement SDGs. Furthermore, some countries lack the infrastructure that is necessary for implementation [27].
8. Culture: Some cultures prohibit people from being open to new ideas and development. This seen significantly with indigenous groups which hampers the implementation of strategies [28].

The timeframe for the SDGs implementation has entered the “Decade of Action” 2020–2030. The UN High Level Political Forum (HLPF) in September 2019 on the “SDG Summit”, stressed that necessity for action on three areas “*global” “local”*, and “*people”* [29].

Table 1, connects the above mentioned SDGs challenges with these areas of actions, and explains some of the governance aspects for each of the challenges.

**Table 1:** Connections between SDG challenges and governance

Global action areas (2020–2030)	SDGs challenges	Governance aspects
Global action	Collective action	International cooperation, political leadership and guidance at international level, political stability
	Accountability	Clear responsibilities to national governments and multi-national organizations
	Financial constrains	Global resources for SDGs, economic equity, private sector finances for SDGs
	Capacity building	Exchange between countries, smart solutions, knowledge transfer
	Technology and data	Integrated global monitoring framework, open data
Local action	Accountability	Clear responsibilities within the public sector entities, scalability to multi-stakeholders and to multi-levels of governance
	Collective action	Organized platforms or cooperation mechanisms that engage a wide range of actors, in national and local level
	Capacity building	Initiatives and resources for capacity building
	Trade-offs	No silos approach and balanced commitment to all SDGs
	Vagueness of goals	Aligning SDGs with national/local agendas and budgets, synergize Targets and Indicators with local national benchmarks
People action	Collective action	Reaching to all layers of society, involving diverse communities in SDGs processes (youth, civil society, media, the private sector, unions, academia etc.)
	Culture	Raise awareness and advocacy about the benefits of SDGs, for different groups of society and individuals
	Financial constrains	Financial resources for communities and individuals to facilitate their commitment to SDGs

The challenges for SDGs are also interdependent. Resolving some issues would result in improving other conditions. For instance, “*Collective action*”, requires guidance and global coordination, which would emphasize inclusive decision making, involvement of diverse actors, and contributions from all sources. On the other hand, enhancing collective action can influence “*Accountability*”, probably by defining clearer roles and responsibilities for the national/local governments, which still remain the main responsible actors. Collective action is also important for “*technology and data*” that would feed to the SDGs framework, through an integrated global approach. Better coordination of financial resources in international level can also resolve some “*financial constraints”*. Exchanges between countries and actors can facilitate “C*apacity building*”.

Additionally, “*Trade*- *offs*” can depend on the counties priorities, and the interests of the donors for specific SDGs. Silos approach from some governments can negatively influence the implementation of the 2030 Agenda as a whole. Uneven implementation of the Goals is also related to unbalance development between countries and their political environment. The ambiguity or “Vagueness” of the Goals can hamper “data and technology” as an obstacle for the SDGs reporting and monitoring framework. Thus risking an unmeasured performance even when there are contributions toward the SDGs. Another important element is the synchronization of different agendas, so the SDGs to become a common language. In this aspect “Culture” and bringing SDGs to the basis would require awareness rising, financial resources and coordination.

## Methodology

Based on the need to foster a better understanding of the consequences of not implementing the SDGs, this work had three main objectives. Firstly, it intended to outline the relevance of the SDGs in achieving global sustainability. Secondly, it aimed to present the main barriers faced in the implementation of the SDGs. Finally, it intended to outline the implications of failing in implementing the 2030 Agenda.

To accomplish those objectives an extended literature review was performed, which is defined by Aboudaoude, Feghali, and Kfouri [30, p. 27], as “a critical look and in depth interpretation and assessment of previous research relevant to the topic or problem being studied”. The literature review was applied to select a comprehensive set of contributions that adequately represent the body of references published within the field of SDGs addressed to its relevance, the state-of-art of its implementation, in terms local and global, as well as, the major challenges reported that hinder its proper implementation and also studies that described the consequences experienced in regions which have had a poor performance in implementation of SDGs.

Several library databases were used in the process of searching and collecting references for this review, including ScienceDirect, Taylor & Francis, Springer, Sage, EBSCO, ProQuest, Wiley, and Emerald.

The following three criteria were employed to select the articles used in this study:

1. Period: studies published between 2012 to 2020;
2. Language: in English;
3. Themes: related to the integration of SDGs and theoretical framework; cases of implementation and performance of SDG in specific regions; major sustainability problems regarding to the low implementation of whole SDGs or a specific objective; advantages and transitions paths related to higher-level implementation of SDG; and, global reporting and monitoring results of SDG implementation.

While contributing to the knowledge on the evolution of SDS implementation over time, the study presented in this article nonetheless is subject to the following limitations: the first is related to the fact that this article only considers references published in English, disregarding cases and reports published in other language then English; the second, considering that the literature on SDGs is exhaustive and full coverage of all the articles is not achievable, there was a need to prioritize the scope considered most globally relevant, and may have disregarded others more specific aspects considered pertinent for a given region.

## Results and discussion

Table 2 presents an overview of some of the consequences of not implementing the SDGs. Due to their importance, the analysis entails all SDGs and does not prioritise any specific one. It can be seen that the consequences of a non-implementation are significant, and may endanger the lives of many people.

**Table 2:** Some of the consequences of not reaching the SDGs. Source: prepared by the authors

SDGs	Implications of not achieving them
	More than half of the world’s population would still have no access to social protection and hundreds of millions would be living in extreme poverty, mostly in Africa [31] If other SDGs would also not be met, conflicts and climate change would worsen the situation. Children would keep being disproportionally affected and it would distress their whole lives [32] If climate action is not taken, extreme weather events would certainly endanger the poverty situation [33]
	Following the tendency currently observed, more people would be undernourished and malnutrition and overweight would still be a serious problem (especially for children) [34] Extreme weather events and conflicts would further threaten food availability [35, 36], affecting also food price. Additionally, food production systems and resilient agricultural practices might not have been implemented, worsening land and soil quality [37] and failing to care for ecosystems
	Local or even global health crisis could be a reality if this goal is not achieved. Spread of epidemics would cause impacts in social life but also in economic issues and supply of medicines, food and water, among other basic needs, demanding a framework of actions for response [38]. The number of deaths and illnesses from the most varied types of risk [39] would demand major investments in treatment interventions
	Not realising this goal would imply in approximately 50% of children in the world without minimum proficiency standards in mathematics and reading and out of school [34], making it more difficult to escape poverty and participate in a tangled global economy (jeopardising also economic growth) Despite the advances in early childhood education, low-income countries would still lack behind and suffer the consequences (primarily related to difficulties for learning at later years) [40]. Lack of investments on lifelong learning and illiterate adults would compromise future opportunities and awareness of the need for behaviour change to more sustainable lifestyles [41]
	Not achieving gender equality would have two main types of consequences: one related to economic issues and other even more worrying, concerning social and physical violence. The former includes unpaid work, denied decision-making positions to women and disproportional salary, among others [42, 43]; the latter refers to millions suffering from forced marriage or physical/sexual violence [44]
	Hundreds of millions would still remain without basic drinking water services and practising open defecation [34]. Clean water and proper sanitation would be unattainable for billions Due to the consequences of not reaching other SDGs as well (including increased infrastructure demand and climate consequences) people could be displaced and experience water stress. This situation would certainly affect education and health [45, 46], among other sustainability goals
	By not increasing considerably the share of renewable energy in the global energy mix, the consequences would be mainly related to climate (e.g. substantial carbon emissions and reduced air quality) [47] in addition to probable conflicts for the consumption of fossil fuels. This might happen especially due to the inefficacy in electrifying the transportation sector [48]. Additionally, billions would still be using polluting cooking systems [34] Despite the positive trend of improvements in energy efficiency, not meeting this target would also prevent the reduction of greenhouse gas emissions [49]. Consequently, increase in temperature and negative effects on climate would be observed
	The current concern of lack of job opportunities, especially for young people [50], and the spread of informal employment would continue to be a preoccupation if this goal is not met. This one might be the SDG with the highest level of disparities among world regions [34]. Unemployment and lack of engagement in education or training could impact other goals [51], worsening poverty and inequality, for example
	The lack of development of manufacturing sectors, especially in developing countries, would endanger productivity and competition [52]. Smaller industries and business would probably not have enough resources to invest in innovation and efficiency This inevitably affects employment and reduction of carbon emissions. Moreover, considering that infrastructure was not deployed to be resilient [53], cities would be at risk to suffer from infrastructure loss or damage
	Inequality within and among countries would imply in prosperity not being shared equally between different population groups [54]. It would negatively affect employment, life quality and probably health as well. If inequality persists in the future, the world would experience more events of migration [55], which expose migrants to several risks and danger in the journey across borders
	The urbanisation represents a challenge to the targets of SDG 11 [56] and if not met it would imply in cities with living conditions that favour carbon emissions and exacerbated use of resources. It pressures waste and water management systems and infrastructure Millions would keep lacking proper waste collection and provision of basic needs (such as sanitation) and living in bad conditions [57]. Harmful air quality, not enough provision of public transportation, and bad network among streets and open public spaces [34] would lead to public dissatisfaction and could cause city crisis
	Not seeing a decoupling of economic growth and natural resources use would imply in bigger global material footprint [58] putting much pressure on the environment. Inappropriate waste management and lack of investments in production and supply chains (i.e. to avoid food waste) would considerably affect availability of resources and pollution of air, water and soil. Detrimental production patterns will also impact greenhouse gas emissions [59]
	Failing in limiting global warming, the concentration of carbon in the atmosphere and the global temperature would entail catastrophic consequences to social systems, to the economy and to the environment, especially due to extreme weather events. Several studies point out these consequences [60, 61], which include sea-level rise and heat waves More resources would be needed to work on mitigation, especially if not invested previously on resilience and adaptation [62, 63]
	Marine life would be endangered due to ocean acidity (that can rise to 100–150% by 2100) [34] and marine pollution. It would also affect the ocean’s role to moderate climate change (attributable to the capacity to absorb carbon dioxide) causing more impacts on water, including sea-level rise By not encouraging sustainable use of oceans and preserving coastal and marine areas, part of marine biodiversity would be in severe risk of extinction [64, 65]
	Not protecting biodiversity and territorial ecosystems would lead species to extinction (or risk of being extinct) [66]. Erosion, deforestation and land degradation would affect millions of people, especially due to loss of essential services of well-being and land productivity [67]. Preservation of biodiversity and different ecosystems is fundamental to climate regulation, so this could be an irreparable loss and worsen extreme weather events
	Not achieving peaceful, just and inclusive societies would prevent millions to experience their security, rights and opportunities [34]. The most serious consequences would be related to risk of murder, sexual exploitation, and forced labour. Conflicts among different territories would also compromise life quality and endanger many lives [68]
	Not realizing this goal might be one of the primary reasons not to achieve the others. Not enough investment and resources would have been mobilised to implement global partnership for the goals [69, 70]. For being the SDG with the highest number of global targets, it complies several approaches for a more sustainable world, including finance, technology, capacity-building, trade and systemic issues, and all of these should have progressed together to achieve the goals

The implications of the non-achievement of the SDGs are significant. For instance, success in achieving poverty reduction is of crucial importance for every country around the world. Even in case of optimistic scenarios, African countries such as Nigeria Benin, Burundi Central African Republic, Malawi, Mali, Mozambique, Somalia, South Sudan, and Zambia are expected to have poverty levels increased by a further 20% by 2030 [71, 72], characterising what is “extreme poverty”. These countries are also likely to be affected by various climate hazards and disasters [72]. Annually, the global economy losses in excess of USD 520 billion means that 26 million additional people are pushed into poverty [73].

Provision of food security is also heavily impacted by climate variability and extreme weather events, threatening the well-being of the poor [74], especially in countries where food needs are not met due to limited resources. In 2017, nearly 821 million people were affected by chronic food deprivation or undernourishment [74].

The global health system is also threatened by spread of infectious diseases. According to latest estimates, total annual losses from a pandemic are about 0.6% of global GDP [75]. The latest EU scenarios project that real GDP growth might fall to 0% or even be considerably negative as a result of the COVID-19 crisis [76]. It is projected that global GDP will decrease by 2.8% in 2020 in comparison to 1.1% in 2009 [77].

Economic development depends also on education that provides children and youth with knowledge and skills necessary for their future. Unfortunately, the quality of children’s’ education not always reach a necessary level. In low-income countries only 20% of all 3- to 6-year-olds have access to preprimary education, globally this value reaches 50% [78]. Despite the increase of the literacy level among youth, there are still 56 and 44 million illiterate women and men between age 5–24 years [79]. Education is facing continuous insufficient funding. In 2012, the share of preprimary education in the education budgets of North America and Western Europe accounted to 8.8%, whereas Sub-Saharan Africa allocated only 0.3% for the same purpose [78]. During the last decade, financing of adult learning and education (ALE) has continued to decrease. Worldwide, 14% of the countries allocate less than 1% to ALE and 19% less than 0.5% [80].

### Non-implementation of the SDGs: a worst-case scenario

The 2019 SDG index offers evidence that not even the most progressed countries topping the index, i.e. the EU’s Nordic countries Sweden, Norway and Finland, managed to enter a transformative path that would lead to truly achieving all the seventeen Sustainable Development Goals by 2030 [81]. Moyer and Bohl [82] warn that many human development related SDG indicators may not be achieved neither by 2030 nor 2050 on the current development trajectory. As global phenomena like climate change, the continued loss of biodiversity and rising inequality are expected to increasingly impact global human development, a new debate that is emerging slowly in the sustainability field centers around the worst-case scenario: What are likely consequences if the implementation of the SDGs falls short, what may happen if the underlying 169 targets are not reached in 2030 by the global community?

Introduced in 2015, the UN Statistical Commission created the Inter-agency and Expert Group on SDG Indicators (IAEG-SDGs) which is continuously working towards improving the global indicator framework. Until today, measuring SDG performance seems a never-ending story, due to the continued need to develop of appropriate methodologies and standards as well as making required country-level data available that allow reliable truly global SDG monitoring and forecasting [83]. As the SDGs are interlinked with each other (see [84]), Donoghue and Khan [85] highlight the critical need to understand synergies and tradeoffs and act accordingly, bearing in mind that decisions taken in one area may negatively endanger progress in others. Sachs et al. [81] point towards substantial negative ecologic and socio-economic spillover effects generated by high-income nations on low-and middle-income nations, e.g. nurturing poor labor standards or promoting deforestation.

The dynamics and complexity of global development are a substantial challenge to forecasting SDGs achievements. Moyer and Bohl [82] argue that achievement of the SDGs should be analyzed at the country, not regional level to capture progresses made. Several approaches can be found in the literature, e.g. using composite SDG indexes (e.g. [81]) that assess and monitor the current state of SDG achievement at the country level and extrapolate results to show trends. Utilizing baseline scenarios such as the Shared Socio-Economic Pathways (SSPs) found in climate science, i.e. cross-cutting projections of a global future (see IPCC), or back-casting or target-seeking scenarios, i.e. exploring how trajectories towards distinctive goals may look like (see, e.g. [86]), in connection with integrated assessment models that allow assessment at the country level, are another way for generating SDG projections [87].

Referring to biodiversity conservation, Kok et al.’s [86] assessment of an ‘option space’ provided by three distinctive pathways (see [88, 89]) for agriculture and forestry, the sectors reported to be responsible for up to 60% of the total reduction in global terrestrial biodiversity, suggested that human development may thrive best if the world pursues a global technology pathway whereas a pathway linked to reduced consumption might be less favorable [82]. However, current forecasts on individual SDG achievement or interacting SDG variables only provide downscaled country-level insights, i.e. possibly diluting developmental differences and resulting in a blurred picture [82, 87].

Despite existing methodological challenges, SDG monitoring and forecasting is progressing and has produced first results that point into similar directions (e.g. [81, 87]. For example, differentiating according to world regions, a current study assumes a ‘middle-of-the-road scenario’ (= SSP2) and analyzing progress of a sub-set of six human development SDGs (i.e. 1, 2, 3, 4, 6 and 7), Moyer and Hedden [87] projected that only 53% of the target indicators assessed may be achieved by 2030. Moreover, 28 particularly vulnerable countries may not achieve any progress in any of the nine human development related target indicators, suggesting an urgent need for initiating action to foster sustainable development within those nations as to leave no one behind.

The dull perspectives suggested by Moyer and Hedden [87] can be underpinned by current monitoring and trends for the respective regions: Presently, Sub-Saharan African countries, show poor performance on socio-economic goals and provide even basic access to services and infrastructure (SDGs 1–9). Negative or even reversed trends are suggested for urban pollution related to sustainable cities (SDG 11), and SDG 15 related to biodiversity loss and deforestation [81]. For the Asia–Pacific region, a recent UN report observed stagnation or even opposite development for more than half the SDGs in the region, especially SDGs 6, 8 and 12. In other areas progress has been achieved, e.g. SDG 1 (poverty), 4 (quality education) and 7 (sustainable energy), but pace appears too slow to meet the 2030 targets [90]. Whereas the situation in East and South Asia may be considered similar relating to SDG 1, 4 and 7 (with exceptions), progress in eight SDGs is slow in most of those nations (SDG 2, 3, 5, 12–15, 16). However, achieving the SDG targets in a region comprising nations if varying sizes and economic development is a complex endeavor. Despite the eradication of poverty seems viable, the targets of SDG13 and 15 will likely not be met in most of East and South Asia on the current trajectory [81, 91]. Progress in most of the SDGs, especially SDG2 (Hunger), SDG 3 (good health and well-being) and SDG 16 (Peace, Justice and Strong Institutions), in the Middle East and North African region is severely impacted where conflicts and wars exist. Achieving SDG2 appears to be a major challenge for all countries in the region [81]. Latin America and the Caribbean lack behind in SDG 12–15 as their economic development negatively affects their environments, and the regions show little progress if at all in SDG 16 due to deadly crime and corruption. Income and wealth inequalities (SDG 10) persist, and access and quality issues relating to health and education impede progress towards SDG 3 and 4 targets [81]. OECD countries’ efforts in terms of climate change mitigation and biodiversity protection need to be accelerated. A lack of agricultural transformation and shifting in diets remains problematic, also inequalities, the poverty of the elderly population and a persistent gender gap relating to pay and unpaid work prevail [81].

This list of consequences is by no means exhaustive, but serves the purpose of illustrating the many implication of lack of concerted action in their implementation. Future research could embrace a qualitative approach, based for instance on a delphi technique, to examine and unveil visions and scenarios from a set selected experts. Other studies may also highlight the implications for diverse types of stakeholders (e.g. policy makers, NGOs, industry, etc.).

## Conclusions

This study has revealed a number of trends. As a start, it can be seen that the list of obstacles to implement the SDGs is long, with many serious problems to be tackled, which at the moment hinder their implementation. But it has also shown that, left unattended, the negative outcomes of not implementing the SDGs may lead to an overall depletion of environmental conditions, an exacerbation of problems such as poverty and hunger.

In addition, economic growth and well-being can also be negatively influenced if a low emphasis to the SDGs is provided. Managing the complexity of SDG implementation is not simple. As they are structured, their implementation depends on partnerships and engagements of local communities and a wide range of stakeholders and require proper sustainability leadership. Moreover, complex issues related to social justice, gender equality and peace are likely to remain unsolved, and many problems will prevail, if the SDGs are not duly implemented.

As this work has shown, the disadvantages of not pursuing the SDGs suggest that much could be gained if they are pursued—and hopefully achieved—so that they are worthy the effort. The implications of this study are twofold. First of all, it sends a clear message about the need to take the SDGs seriously, and pay better attention to the many factors which affect their implementation. Secondly, the original analysis of the implications of not achieving the SDGs, as provided here, should be seen as a warning sign.

In terms of implications for further research, this paper outlines the need for a greater emphasis on a critical analyses and empirical assessments of the degree to which the 17 SDGs are being implemented. It may also pave the way for further studies which may investigate the consequences to specific sectors (e.g. agriculture) and population groups (e.g. farmers, herders, fishing communities) whose livelihoods heavily depend on nature, natural resources and ecosystem services.

A failure to achieve the SDGs is likely to negatively affect billions of people round the world, with substantial damages to livelihoods, an exacerbation of poverty and the spread of diseases. These, in turn, will particularly affect people in developing countries, which are especially vulnerable.
